# Highly Efficient, Rapid, and Simultaneous Removal of Cationic Dyes from Aqueous Solution Using Monodispersed Mesoporous Silica Nanoparticles as the Adsorbent

**DOI:** 10.3390/nano8010004

**Published:** 2017-12-23

**Authors:** Peige Qin, Yixin Yang, Xiaoting Zhang, Jiahua Niu, Hui Yang, Shufang Tian, Jinhua Zhu, Minghua Lu

**Affiliations:** 1 Institute of Environmental and Analysis Science, School of Chemistry and Chemical Engineering, Henan University, Kaifeng 475004, Henan, China; qinpeige@outlook.com (P.Q.); yangyixin7375@outlook.com (Y.Y.); zhangxiaoting1990@yahoo.com (X.Z.); niujiahua@outlook.com (J.N.); tianshufang@henu.edu.cn (S.T.); zhujinhua@henu.edu.cn (J.Z.); 2 Institute of Pharmacy, Pharmaceutical College, Henan University, Kaifeng 475004, Henan, China; yanghui_wg@henu.edu.cn

**Keywords:** adsorption, cationic dyes, dye removal, monodispersed mesoporous silica nanoparticles

## Abstract

In this work, a highly efficient and rapid method for simultaneously removing cationic dyes from aqueous solutions was developed by using monodispersed mesoporous silica nanoparticles (MSNs) as the adsorbents. The MSNs were prepared by a facile one-pot method and characterized by scanning electron microscopy, transmission electron microscopy, Fourier-transform infrared spectroscopy, and Brunauer-Emmett-Teller. Experimental results demonstrated that the as-prepared MSNs possessed a large specific surface area (about 585 m^2^/g), uniform particle size (about 30 nm), large pore volume (1.175 cm^3^/g), and narrow pore size distribution (1.68 nm). The materials showed highly efficient and rapid adsorption properties for cationic dyes including rhodamine B, methylene blue, methyl violet, malachite green, and basic fuchsin. Under the optimized conditions, the maximum adsorption capacities for the above mentioned cationic dyes were in the range of 14.70 mg/g to 34.23 mg/g, which could be achieved within 2 to 6 min. The probable adsorption mechanism of MSNs for adsorption of cationic dyes is proposed. It could be considered that the adsorption is mainly controlled by electrostatic interactions and hydrogen bonding between the cationic dyes and MSNs. As a low-cost, biocompatible, and environmentally friendly material, MSNs have a potential application in wastewater treatment for removing some environmental cationic contaminants.

## 1. Introduction

Nowadays, more than 100,000 commercial dyes are used in various fields, such as the textiles, paper, printing, rubber, plastics, cosmetics, leather tanning, food processing, and dye manufacturing industries [1,2]. It is known that about 10%–15% of all dyes used in the industry are lost in the wastewater during processing [3]. Because many dyes are carcinogenic, mutagenic, and teratogenic compounds, dye-containing wastewater not only contaminates surface and groundwater but also harms human health and disrupts the ecological system [4,5,6]. Owing to their high thermal and chemical stability, many dyes are resistant to degradation by light, heat, and oxidants in nature [7]. Therefore, the removal of dyes from wastewater has become a significant issue worldwide. 

Up to now, many approaches have been developed to deal with dye contaminants in wastewater, including adsorption [8,9], flocculation [10], coagulation [11], electrolysis [12], biodegradation [13], and photocatalytic degradation [14,15,16]. Among these methods and techniques, adsorption is considered to be a promising strategy due to its inherently high efficiency, economic feasibility, good biocompatibility, and simplicity in operation [6,17,18]. In recent years, various materials (e.g., activated carbons [19], ordered mesoporous carbons [20,21,22,23], carbon nanotubes [24], graphene-based nanocomposites [25,26], metallogels [27], metal-organic frameworks [7,28,29], and organosilica nanoparticles [30]) have been used as adsorbents for the adsorption of dyes from wastewater. However, most of these adsorbents are not widely used because of high cost, poor selectivity, complex preparation processes, and difficult disposal [31]. Hence, it is important to find a highly efficient and economic adsorbent with high selectivity and short contact time toward organic dyes.

Since first introduced in the 1990s by Kresge [32], mesoporous silica nanoparticles (MSNs) have attracted much attention because of their intrinsic mesostructural properties, such as high specific surface area, large pore volume, and controllable and uniform particle size [33]. Due to their chemical inertness and excellent biocompatibility, MSNs and surface-functionalized MSNs exhibit vast potential application in the adsorption of heavy metal ions and organic compounds, as well as for the delivery of various drug molecules and biomolecules (e.g., proteins, DNA, genes, and enzymes) [34,35,36,37,38]. MSNs have also been demonstrated to be an excellent adsorbent for removing dyes from aqueous media [39]. Highly-ordered mesoporous SBA-15 molecular sieves were synthesized and applied for the adsorption of cationic dyes such as methylene blue (MB) and Janus Green B [40]. SBA-16 [41] and Si-MCM-41 [42] materials have been reported as an excellent adsorbent for adsorption of cationic, neutral, and anionic dyes. Tsai et al. [43] synthesized cubic mesoporous silica SBA-16functionalized with carboxylic acid for effective removal of cationic dyes. Recently, Liang and coworkers [44] prepared CuO/meso-silica nanocomposite for further enhancing the adsorption ability of meso-silica MCM-41 towards dyes.

**Figure 1:**
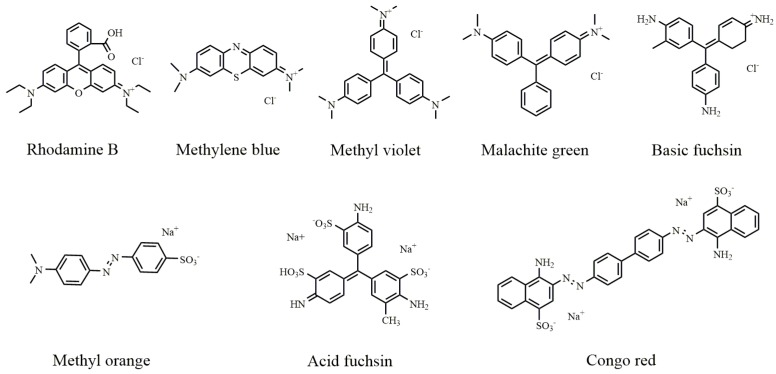
Chemical structure of cationic and anionic dyes mentioned in this study.

**Scheme 1:**
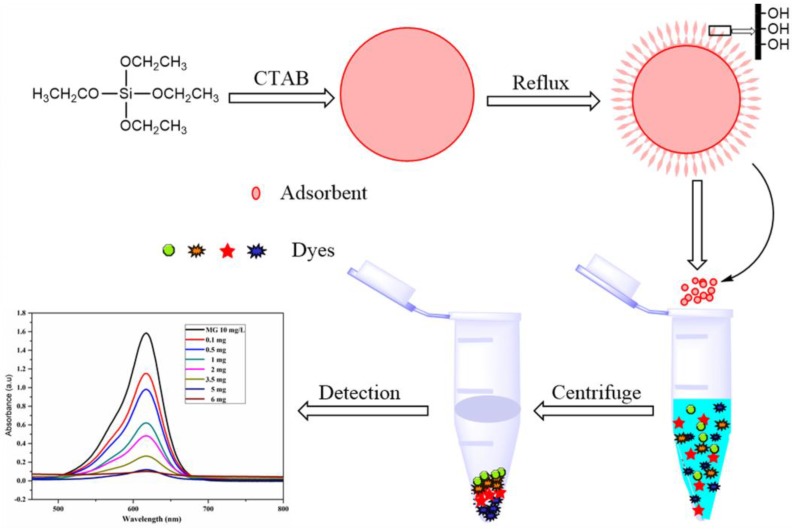
Schematic illustration for the preparation of monodispersed MSNs used as the adsorbent for the removal of cationic dyes from aqueous solution.

In this study, we demonstrated a simple and rapid approach for simultaneously removing cationic dyes (chemical structure is shown in Figure 1) including rhodamine B (RhB), methylene blue (MB), methyl violet (MV), malachite green (MG), and basic fuchsin (BF) from aqueous solutions by using monodispersed MSNs as the adsorbent (Scheme 1). Compared with other adsorption materials that usually require very long adsorption time (usually a few hours), the adsorption capacities of the proposed material for the above mentioned cationic dyes in the range of 14.70 mg/g to 34.23 mg/g could be achieved within 2 min to 6 min. As a low-cost, environmentally friendly, and good biocompatible material, it could be considered that monodispersed MSNs would have a potential application for the removal of cationic dyes from wastewater.

## 2. Materials and Methods

### 2.1. Materials

Cetyltrimethyl ammonium bromide (CTAB, 99%) and tetraethyl orthosilicate (TEOS, 98%) were obtained from Aladdin (Shanghai, China). N,N,N,N-Tetrakis(2-hydroxyethyl)ethylenediamine (THEED, 99%) was purchased from Acros Organics (Geel, Belgium). Methylene blue (MB, C_16_H_18_ClN_3_S), methyl violet (MV, C_25_H_30_ClN_3_), basic fuchsin (BF, C_20_H_20_ClN_3_), malachite green (MG, C_23_H_25_ClN_2_), methyl orange (MO, C_14_H_14_N_3_NaO_3_S), and acid fuchsin (AF, C_20_H_17_N_3_Na_2_O_9_S_3_) were supplied by Tianjin Kemiou Chemical Reagent Co., Ltd. (Tianjin, China). Rhodamine B (RhB, C_28_H_31_ClN_2_O_3_) and Congo red (CR, C_32_H_22_N_6_Na_2_O_6_S_2_) were obtained from Shanghai Chemical Reagent Co., Ltd. (Shanghai, China). Ultrapure water was produced by a Millipore Milli-Q ultrapure water system (Millipore, Bedford, MA, USA). All the chemicals were used without further purification.

### 2.2. Synthesis of MSNs

Monodispersed MSNs were prepared according to the literature [45] with some modifications described as follows. Briefly, 153.5 mg of CTAB and 43.0 mg of THEED were dissolved in 10.0 mL of ultrapure water by ultrasonic treatment. The obtained homogeneous clear solution was then heated to 60 °C for 30 min under magnetic stirring (1000 r/min). Then, 1.2 mL of TEOS was added drop by drop to the dispersion with vigorous stirring (1500 r/min); stirring of the dispersion was continued for 0.5 h at 60°C.Next, 0.3 mL of TEOS was added dropwise and the dispersion was further stirred at 60°C for 2.5 h. After the reaction, all the products were collected by centrifugation and washed several times with water and ethyl alcohol. In order to remove the CTAB template, the obtained white powder was dispersed in a mixed solution of 80 mL ethanol (10.0 mg/L of ammonium nitrate solution) and refluxed at 80°C for 10 h. In the end, the microspheres were washed with ultrapure water and dried at 60 °C for 12 h; the MSN microspheres were finally synthesized.

### 2.3. Adsorption Experiments

The adsorption performance of MSNs was investigated by adsorbing cationic dyes including RhB, MB, MV, MG, and BF and anionic dyes including AF, MO, and CR from aqueous solution in batch mode. The stock dye solution with a concentration of 1.0 g/L was prepared by dissolving the corresponding dye in ultrapure water. A series of working solutions with varying concentrations were prepared from the stock solution with ultrapure water. Then, 5.0 mg MSNs were added to 26 mL deionized water in 50 mL brown glass tubes, and certain volumes of the dye stock solutions were added to give initial concentrations reaching 10 mg/L. The pH was adjusted to 7.0 with NaOH (0.01 M) or HCl (0.01 M). The final volumes of the solutions were adjusted to 30 mL with deionized water, and the tubes were shaken in a magnetic stirrer (IKA^®^RCT, Baden-Württemberg, Germany) at 130 r/min at 25 °C. After adsorption for a predetermined time, the mixtures were centrifuged for 3 min at 11,000 rpm and the supernatants were collected. The concentration of the residual dyes in the supernatant was determined by measuring the absorbance of the solution at maximum wavelength with a TU-1900 dual-beam UV-Vis spectrophotometer (Puxi General Instrument Co., Ltd., Beijing, China) at room temperature.

The adsorption properties of the MSNs for the dye solutions were investigated using a contrast design. The adsorption amount and adsorption rate (percentage removal) of the dye on MSNs were calculated by the following equation:

(1) $$q=[(C_{0}-C)\times V]$$

(2) $$Sorption\left(\%\right)=\left[\left(C_{0}-C\right)/C_{0}\right]\times 100\%$$

where $C_{0}$ and C (mg/L) are the concentrations of the dye solution before and after sorption, respectively. q is the amount of cationic dye (mg) absorbed on the adsorbent, V (L) is the volume of the dye solution.

### 2.4. Desorption Experiments

For the desorption study, the cationic dye was adsorbed on MSN material under the optimized adsorption conditions. The MSN material with adsorbed corresponding dye was used for the desorption study. Ethanol was used for regeneration of the MSN adsorbent. To determine the reusability of the MSN material, four successive adsorption–desorption cycles were performed. The concentrations of the desorbed dyes in the supernatants were determined by measuring the absorbance of the solutions at maximum wavelength with a TU-1900 dual-beam UV-Vis spectrophotometer at room temperature. The percentage desorption was calculated by the following equation:

(3) $$Desorption\;(\%)=\frac{\begin{array}{cc} Concentration & desorbed\left(mg/L\right) \end{array}}{\begin{array}{cc} Concentration & desorbed\left(mg/L\right) \end{array}}\times 100\%$$

### 2.5. Material Characterization

The morphology of the MSNs was observed using scanning electron microscopy (SEM, JSM-7610F, Tokyo, Japan) with an accelerating voltage of 10 kV. Transmission electron microscopy (TEM) was achieved on a JEM 2100 (Osaka, Japan). The samples for TEM characterization were prepared by placing a drop of a colloidal solution on a carbon-coated copper grid, which was dried at room temperature. Fourier-transform infrared (FT-IR) spectra were collected on an infrared Fourier-transform spectrometer using KBr pellets (VERTEX 70, Bruker, Karlsruheb, Germany) within the wavelength range 4000–500 cm^−1^. The Brunauer-Emmett-Teller (BET) surface area of the MSNs was determined by N_2_ adsorption–desorption isotherms (ASAP 2020, Micromeritics, USA). The zeta potentials of samples suspended in aqueous solution were measured using a Malvern Instrument nanoZS (Worcestershire, UK) based on the method of an electrophoretic light scattering technique that measures the migration rate of dispersed particles under the influence of an electric field. Suspensions of samples in ultrapure water were prepared at various pH. After five times of measurements, the average value and standard deviation of the zeta potential were recorded. The size distribution of MSNs was also determined by dynamic light scattering (DLS, Malvern Zetasizer Nano ZS, Worcestershire, UK).

### 2.6. Dye Concentration Measurement

The concentrations of the dye samples were determined by using a UV-Vis spectrophotometer (TU-1900, Puxi General Instrument Co., Ltd., Beijing, China). The calibration curve was acquired for each dye using eight concentrations of standard dye solutions and the concentrations of the dyes ranged typically from 0.1 mg/L to 15.0 mg/L. This step was repeated three times to ensure the repeatability of the calibration curve, and all of the dyes exhibited satisfactory linearity with correlation coefficients (*R*^2^) above 0.9964. 

## 3. Results and Discussion

### 3.1. Characterization of MSNs

**Figure 2:**
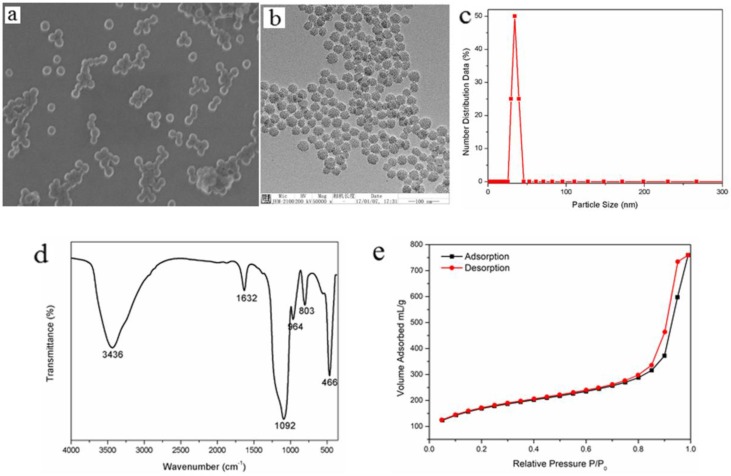
Scanning electron microscopy (SEM) image (**a**), transmission electron microscopy (TEM) image (**b**), particle size distribution (**c**), Fourier-transform infrared (FT-IR) spectra (**d**), and nitrogen adsorption-desorption isotherm of the as-prepared monodispersed mesoporous silica nanoparticles (MSNs) (**e**).

The morphology and particle size of the as-prepared MSNs were investigated by SEM and TEM. As shown in Figure 2a,b, the SEM and TEM images demonstrate that all the MSNs exhibited spherical morphologies with a uniform particle size (about 30 nm). A similar conclusion about the particle sizes of MSNs could also be achieved from Figure 2c. Moreover, TEM results confirmed the existence of pores on the MSN surfaces.

Figure 2d shows the FT-IR spectra of the MSNs. The obvious characteristic absorption peaks can be seen at 3436 cm^−1^, 1632 cm^−1^, 1092 cm^−1^, 964 cm^−1^, 803 cm^−1^, and 466 cm^−1^ and of these the peak at 466 cm^−1^ is attributed to the bending vibration of O–Si–O, the peak at 1092 cm^−1^ corresponds to Si–O–Si stretching vibration, and the peaks at 964 cm^−1^ and 1632 cm^−1^ correspond to the Si–OH bending vibration. The broad absorption peak at 3436 cm^−1^ isdue to O–H stretching vibration, which can be assigned to the water molecules. All the mentioned spectral data confirm that the MSNs were successfully synthesized and have a large number of hydroxyl groups on the nanomaterial surface.

The specific surface area and the pore volume are the main factors influencing the adsorption capacity. The N_2_ adsorption-desorption isotherms were used to measure the porosity of the as-prepared MSNs. As shown in Figure 2e, N_2_ adsorption-desorption isotherms of MSNs exhibited typical type IV curves with a sharp uptake at a high relative pressure (*P*/*P*_0_ > 0.8), which demonstrates the existence of voids between particles. The surface area, total pore volume, and pore size of the MSNs were determined as 584.98 m^2^/g, 1.175 cm^3^/g, 1.68 nm by density functional theory (DFT) method, respectively.

### 3.2. Optimized Adsorption Conditions

#### 3.2.1. Effect of Adsorbent Mass

**Figure 3:**
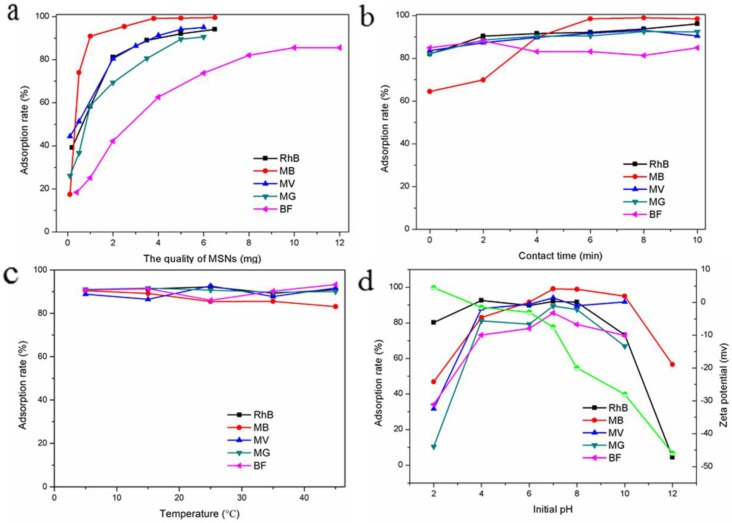
The effect of the mass of the adsorbent (**a**), the contact time (**b**), the temperature (**c**) and the initial pH (**d**) on the adsorption efficiency.

The effect of the adsorbent mass in the range of 0.2 mg to 12.0 mg on the adsorption rate was investigated first. As shown in Figure 3a, the adsorption rate increased gradually with an increase in the adsorbent mass from 0.2 mg to 5 mg, and then changed slowly after that. However, the adsorption rate of MSNs towards BF reached 83% at 10.0 mg. Therefore, in the following experiments, 5.0 mg of the MSN material was used for dyes except BF (10.0 mg).

#### 3.2.2. Effect of Contact Time

Contact time is another important factor that influences the adsorption efficiency. The contact time was studied in the range of 0 to 10 min with other experimental conditions being held at fixed values. Figure 3b shows the effect of contact time on the adsorption ratio of dyes. It can be concluded that the adsorption process reached an equilibrium state within 6 min for all of the dyes and then changed slowly. Therefore, 6 min was selected as the optimized contact time for five dyes including RhB, MV, MG, MB, and BF.

#### 3.2.3. Effect of Temperature

The temperature effects on adsorption efficiency was investigated between 5 °C and 45 °C, and the experimental results are demonstrated in Figure 3c. It can be seen that the adsorption rate kept constant with increasing temperature; this may be because temperature has little effect on the adsorption efficiency. Therefore, room temperature (about 25 °C) was selected for this experiment.

#### 3.2.4. Effect of Initial pH

The initial pH is one of the most important parameters in the adsorption process because it can affect the interaction of the surface functional groups of the adsorbent. The effects of the initial pH on adsorption were studied over a pH range of 2.0–12.0 for RhB and MB, and over a pH range of 2.0–10.0 for the other dyes. HCl and NaOH were used to adjust the pH of the dye solutions. Figure 3d shows the adsorption rate for the cationic dyes on the surface of MSNs with different pH. The results indicate that the adsorption capacity of MSNs was strongly dependent on the pH value of the solution. As can be seen from Figure 3d, the adsorption rate increased with an increasing pH value from 2.0 to 7.0. However, when the pH value was changed from 7.0 to 12.0, the adsorption rate decreased. This phenomenon can be attributed to the adsorption of cationic dyes on MSNs being a hydrogen bond controlled adsorption. In the low pH solution, a hydrated proton (H_3_O^+^) can be combined with hydroxyl groups to form an interaction, which restrains the hydrogen bond interaction between the cationic dyes and MSNs [7]. However, in an alkaline solution, the hydroxyl groups may combine with the amino groups on the cationic dyes to form NH_3_·H_2_O. Therefore, the adsorption rate decreased with increasing pH values from 7.0 to 10.0 or 12.0 since the hydrogen bond interaction between the cationic dyes and MSNs would be weakened in alkaline solution.

On the other hand, the adsorption can be explained by electrostatic interactions between the negatively charged surface of MSNs and the positively charged cationic dyes. The zeta potential of the absorbent is one of the important factors that affect the adsorption capacity. The surface charge of the adsorbent was characterized by pH_pzc_ (point of zero charge). In aqueous solutions, when pH < pH_pzc_, the surface charge of MSNs is positive, while it is negative when pH > pH_pzc_. The pH_pzc_ of MSNs is shown in Figure 3d. At pH = 2, the surface of MSNs is positively charged, while at pH > 2, the surface is negatively charged. Meanwhile, RhB, MB, MV, MG, and BF as the cationic dyes dissociate to chloride ions (Cl^−^) and amino cations (R_2_-N^+^) in aqueous solution. In the range of the pH above pH_pzc_, the MSNs surface carries negative charges, which benefits the adsorption of cationic dyes onto MSNs through electrostatic interaction. Therefore, the adsorption rates were improved with changing pH values from 2.0 to 7.0 since the number of negatively charged sites were increased with increasing pH values at this range. When pH values were higher than 7.0, the adsorption rates of MSNs for cationic dyes decreased with further increasing pH values. This can be ascribed to the hydrogen bond interaction between cationic dyes and MSNs, which is stronger than that of an electrostatic interaction. Therefore, pH 7.0 was selected for the following experiments.

#### 3.2.5. Adsorption Isotherms

**Figure 4:**
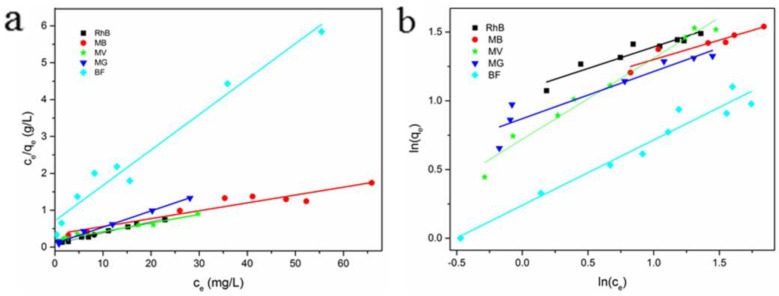

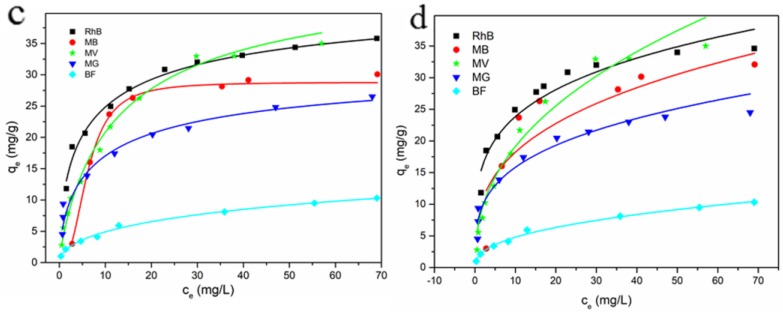
Adsorption isotherm models of MSNs for adsorption of cationic dyes with the Langmuir model (**a**), Freundlich model (**b**), and plot of adsorption capacity (*q_e_*) vs. equilibrium concentration of MSNs for adsorption of cationic dyes according to the Langmuir model (**c**).

In this work, both Langmuir and Freundlich models were used to explain the experimental results. The Langmuir model is assumed for ideal monolayer adsorption. The adsorption isotherm is based on the assumption that there are fixed numbers of active sites on the surface of the adsorbent. Each site is available for only one adsorbate molecule to occupy it. Once an adsorbate molecule occupies a site, no further adsorption can take place at that site, with no interaction between adsorbed species. The linear equation of Langmuir adsorption can be expressed as:

(4) $$q_{e}=\frac{K_{L}C_{e}q_{m}}{1+K_{L}C_{e}}$$

(5) $$\frac{C_{e}}{q_{e}}=\frac{1}{q_{m}\times K_{L}}+\frac{1}{q_{m}}\times C_{e}$$

where $C_{e}$ is the equilibrium concentration of the dye in the aqueous solution (mg/L), $q_{e}$ is the equilibrium amount of the cationic dye adsorption by MSNs (mg/g), $q_{m}$ is the theoretical dye maximum adsorption capacity (mg/g), and $K_{L}$ is the Langmuir isotherms constant (L/mg). Figure 4a shows the plot of $\frac{C_{e}}{q_{e}}$ vs. $C_{e}$, Figure 4c shows the plot of $q_{e}$ vs. $C_{e}$, and values of the parameters are listed in Table 1.

The Freundlich model is another frequently applied isotherm model. Usually, it is applied in describing heterogeneous systems and is characterized by a heterogeneity factor of *n*. Our data were fitted to the Freundlich isotherm model, and the linear equation is expressed as follows:

(6) $$\ln q_{e}=\ln K_{F}+\frac{1}{n}\times \ln C_{e}$$

where *K_F_* indicates the Freundlich constant and *n* (dimensionless) is the heterogeneity factor. When 0 < 1/*n* < 1, the adsorption is favorable; when 1/*n* = 1, the adsorption is irreversible; and when 1/*n* > 1, the adsorption is unfavorable. Figure 4b illustrates the plot of $\ln q_{e}$ vs. $\ln C_{e}$, Figure 4d illustrates the plot of $q_{e}$ vs. $C_{e}$, and the values of the parameters are given in Table 1.

It can be concluded from Figure 4 that the Langmuir isotherm model showed better mathematical fit with the experimental data than the Freundlich isotherm model (based on the higher correlation coefficient (*R*^2^)). The result indicates that the Langmuir model is suitable for describing the adsorption equilibrium of cationic dyes onto MSNs.

#### 3.2.6. Adsorption Mechanism Prediction

According to the analysis of the effect of pH and adsorption isotherms, it can be explained that the adsorption of cationic dyes is related to the chemical action between cationic dyes and MSNs. Considering the chemical structure of the adsorbent surface, as well as the difference in the chemical structures of the cationic and anionic dyes, hydrogen bonds could occur between the hydroxyl surface groups of the MSNs as proton donors and the nitrogen atoms (R_2_-NH) in the cationic dye molecules as proton acceptors. On the other hand, according to the discussion above and the zeta potential, the adsorption mechanism can be explained by electrostatic interactions between the positively charged cationic dyes and the negatively charged surface of the MSNs. 

### 3.3. Adsorption Selectivity

**Figure 5:**
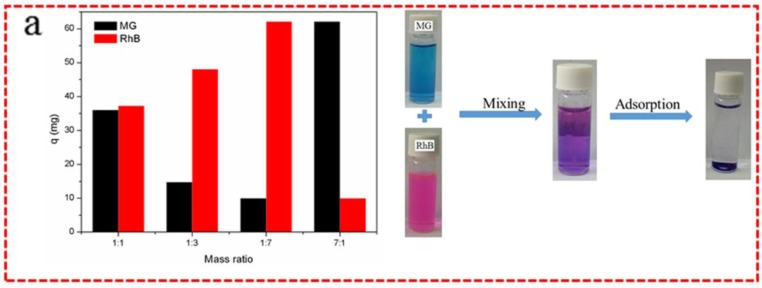

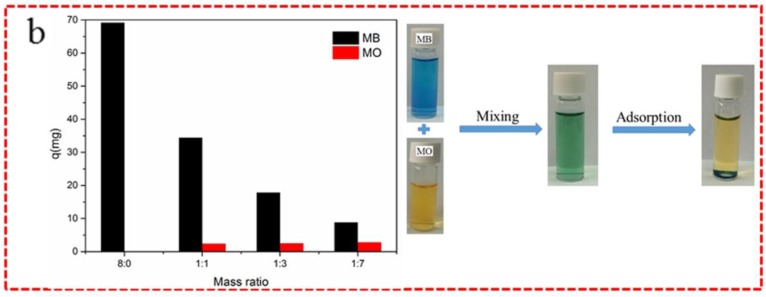
Simultaneous adsorption of two cationic dyes (malachite green (MG) and rhodamine B (RhB)) with the same concentration (10.0 mg/L) at different mass ratios (**left**) and their photographs at same ratio (**right**) (**a**), and simultaneous adsorption of one cationic dye (methylene blue (MB)) and one anionic dye (methyl orange (MO)) with the same concentration (10.0 mg/L) at different mass ratios (**left**) and their photographs at same ratios (**right**) (**b**).

To investigate the selectivity of the as-prepared monodispersed MSNs for different dyes, 8.0 mL solution containing two cationic dyes (MG and RhB) was first selected. The initial volume ratios of MG and RhB in the mixture (initial concentration is 10.0 mg/L) were set as 1:1, 1:3, 1:7, 7:1, respectively. After being still placed for 6 min and centrifugation, the mixture solution was determined and the result is shown in Figure 5a. It can be seen that the two cationic dyes were adsorbed at the same time, which indicates that the adsorption of MSNs for cationic dyes is a noncompetitive adsorption. Another solution containing one cationic dye (MB) and one anionic dye (MO) was studied. MSNs (5.0 mg) were added to 8.0 mL of above solution with the same concentration of 10.0 mg/L for MB and MO. The initial volume ratios of MB and MO were set as 8:0, 1:1, 1:3, 1:7, respectively. After being still placed for 6 min and centrifugation, the solution was determined and the result is demonstrated in Figure 5b. It can be concluded that the materials could selectively adsorb cationic dyes from the solution. 

### 3.4. Repeatability Study

**Figure 6:**
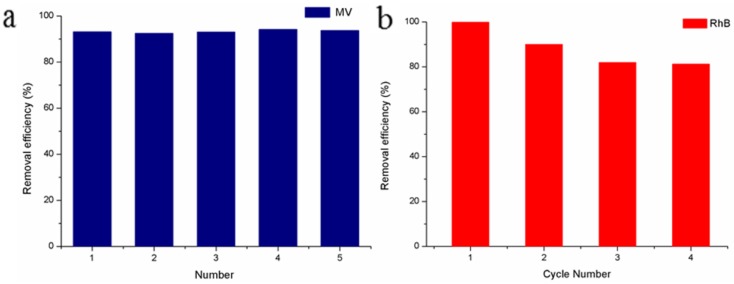
Repeatability (**a**) and reusability (**b**) of MSNs for adsorption of cationic dyes.

A promising adsorbent should have a steady adsorption capacity in parallel experiments. The repeatability of the MSNs for adsorption of cationic dyes was investigated by analyzing five samples containing the same concentration of cationic dye and the result is illustrated in Figure 6a. The relative standard deviation (RSD) for five parallel analyses was 0.62%, which indicates that the materials have a stable adsorption. 

### 3.5. Reusability Study

Recycling and reuse are of great importance for adsorbents in practical applications. Therefore, RhB was selected to study the reusability of MSNs. After adsorption of RhB, the MSNs-RhB was immersed in ethanol with ultrasonic extraction for 10 min and repeated several times. Then, the adsorbents were dried for further using. As can be seen from Figure 6b, the desorption rate of MSNs for RhB remained above 80% after four consecutive adsorption/desorption cycles. It can be concluded that the as-prepared monodispersed MSNs have a good reusability for removing cationic dyes in aqueous solution.

### 3.6. Comparison with Other Absorbents

To evaluate the performance of the MSNs for the adsorption of cationic dyes, other frequently used absorbents were selected for comparison. The results are presented in Table 2. Compared with other silica adsorbents that usually require one hour or more, the MSNs as an adsorbent for removing cationic dyes finished in several mins (2 min to 6 min) with satisfactory adsorption capacity. As a low-cost, biocompatible, and environmentally friendly material, MSNs have a potential application in wastewater treatment for removing some environmental contaminants.

## 4. Conclusions

In this work, monodispersed MSNs were prepared in a simple way and its adsorption of five cationic dyes was investigated. The results show that the MSNs exhibited a rapid and selective adsorption ability towards cationic dyes instead of an anionic dye (MO) in aqueous solutions. The as-prepared monodispersed MSNs exhibited satisfactory adsorption efficiency (14.7 to 37.32 mg/g) and rapid adsorption properties (2 min to 6 min). The adsorption of cationic dyes by monodispersed MSNs might be attributed to chemical interactions, including hydrogen bonding, and physical adsorption to the surface of materials. As an economic and environmental material, monodispersed MSNs might have a potential application in the treatment of wastewaters containing cationic dyes.

## Figures and Tables

**Table 1:** Langmuir and Freundlich parameters for the adsorption of cationic dyes onto MSNs at 25 °C.

Dyes	Langmuir Model			Freundlich Model		
	*q_m_* (mg/g)	*K_L_* (L/g)	*R*^2^	*K_F_* (L/g)	1/*n*	*R*^2^
RhB	33.22	0.3500	0.9890	9.330	0.6900	0.9432
MB	38.17	0.098	0.9731	10.39	0.2800	0.8904
MV	40.65	0.1300	0.9793	5.260	0.5800	0.9747
MG	22.68	0.444	0.9940	4.445	0.3400	0.8874
BF	10.41	0.1350	0.9666	1.736	0.4700	0.9544

**Table 2:** Comparison of the maximum adsorption capacities (*Q*_max_, mg/g) of other reported silica materials and MSN in this work.

Adsorbents	Contact Time (min)	Dyes	*Q*_max_ (mg/g)	Reference
CCMSN	300	Methylene blue	43.03	[39]
SBA-15	60	Methylene blue Janus Green B	49.26 66.44	[40]
Si-MCM-41	30	Safranin O	275.5	[41]
SBA-16	30	Safranin O	240.39	[42]
S16C-30 CuO/MCM-41	200 60	Methylene blue Methylene blue	561 87.8	[43] [44]
MSNs	2~6	Methylene blue Rhodamine B Methyl violet Malachite green Basic fuchsin	34.23 23.26 20.36 20.10 14.70	This work

## Abbreviations

MSNs	mesoporous silica nanoparticles
RhB	rhodamine B
MB	methylene blue
MV	methyl violet
MG	malachite green
BF	basic fuchsin
AF	acid fuchsin
MO	methyl orange
CR	congo red
CTAB	cetyltrimethyl ammonium bromide
TEOS	tetraethyl orthosilicate
THEED	N,N,N,N-Tetrakis (2-hydroxyethyl)ethylenediamine
SEM	scanning electron microscopy
TEM	transmission electron microscopy
FT-IR	Fourier-transform infrared
BET	Brunauer-Emmett-Teller
DFT	Density functional theory
*R*^2^	correlation coefficients
pH_pzc_	point of zero charge
